# Influence of a ketogenic diet, fish-oil, and calorie restriction on plasma metabolites and lipids in C57BL/6J mice

**DOI:** 10.1186/1743-7075-11-23

**Published:** 2014-05-22

**Authors:** Joshua J Meidenbauer, Nathan Ta, Thomas N Seyfried

**Affiliations:** 1 Biology Department, Boston College, Chestnut Hill, MA 02467, USA

**Keywords:** Ketogenic diet, Omega-3 fatty acids, Calorie restriction, Glucose, Ketones

## Abstract

**Background:**

Diet therapies including calorie restriction, ketogenic diets, and fish-oil supplementation have been used to improve health and to treat a variety of neurological and non-neurological diseases.

**Methods:**

We investigated the effects of three diets on circulating plasma metabolites (glucose and β-hydroxybutyrate), hormones (insulin and adiponectin), and lipids over a 32-day period in C57BL/6J mice. The diets evaluated included a standard rodent diet (SD), a ketogenic diet (KD), and a standard rodent diet supplemented with fish-oil (FO). Each diet was administered in either unrestricted (UR) or restricted (R) amounts to reduce body weight by 20%.

**Results:**

The KD-UR increased body weight and glucose levels and promoted a hyperlipidemic profile, whereas the FO-UR decreased body weight and glucose levels and promoted a normolipidemic profile, compared to the SD-UR. When administered in restricted amounts, all three diets produced a similar plasma metabolite profile, which included decreased glucose levels and a normolipidemic profile. Linear regression analysis showed that circulating glucose most strongly predicted body weight and triglyceride levels, whereas calorie intake moderately predicted glucose levels and strongly predicted ketone body levels.

**Conclusions:**

These results suggest that biomarkers of health can be improved when diets are consumed in restricted amounts, regardless of macronutrient composition.

## Background

Mounting evidence suggests that dietary intake can influence the prognosis of a broad range of diseases, including epilepsy, autism, cancer, Alzheimer’s disease, and cardiovascular disease [1-5]. Popular diets to treat these conditions include the ketogenic diet, low-glycemic index treatment diet, fish-oil supplemented diets, and calorie restricted diets. There is continued debate and interest on how dietary composition and quantity affects body weight, blood lipid profile, and glycemic control [6-10]. In order to effectively utilize dietary intervention to treat disease, it is important to understand how different treatment modalities could affect plasma metabolites, such as glucose and ketone bodies, and the overall health and vitality of the subjects.

Plasma glucose and ketone levels are important prognosticators of dietary efficacy for a variety of neurological and non-neurological disorders [5,11-16]. In epilepsy, studies revealed that the efficacy of the restricted ketogenic diet relies on how well the diet lowers blood glucose and elevates blood ketone levels [17-19]. Similarly, preclinical and clinical studies have shown a positive effect of increased circulating ketone levels and reduced glucose levels in disease outcome in cancer [13,20-22]. In healthy individuals, however, it is unclear whether reduced glucose levels and increased ketone levels improves health, though it may be associated with metabolic disease resistance [23].

Conflicting results on blood lipid and glucose levels were reported in humans and mice on the ketogenic diet. In adults on the ketogenic diet, triglyceride levels remain unchanged or are lowered, whereas cholesterol levels remain unchanged, are lowered, or are increased [24-27]. The ketogenic diet increases plasma triglyceride and cholesterol levels in children [28]. Fasting glucose in both adults and children remains unchanged or is lowered on the ketogenic diet [27,29-33], whereas the blood ketones are consistently increased [34]. In mice, the ketogenic diet decreases plasma triglyceride levels while increasing cholesterol levels [35,36]. Increased, decreased, or no change in plasma glucose levels were reported in mice on the ketogenic diet [13,18,35]. As in children, plasma ketone levels are consistently elevated in mice on ketogenic diets [18,35].

Supplementation with fish-oil leads to more consistent effects on blood lipid and glucose levels, compared to the ketogenic diet. Blood triglyceride levels decrease, whereas total cholesterol levels remain unchanged in humans [37-41]. Fasting glucose levels are unchanged in non-diabetic individuals, but are increased in type-2 diabetic individuals that supplement with fish-oil [39-41]. Blood ketone levels remain unchanged after supplementation with fish-oil [42]. Fish-oil supplemented and derived diets in mice reduce total plasma cholesterol and either decrease or have no effect on plasma triglyceride levels [43-45]. Fasting glucose in mice remains unchanged or is decreased in mice given fish-oil supplemented or derived diets [44-46]. Hepatic ketogenesis increased in rats fed a fish-oil-supplemented diet, due to decreased lipogenesis and increase in fatty acid oxidation [47].

Calorie restriction is a long-studied dietary modality that is often used in conjunction with the ketogenic diet to treat a variety of neurological disorders [48], and is used as a prophylactic for cardiovascular disorders and other pathologies [49-51]. Calorie restriction in humans leads to consistent reductions in total cholesterol and triglyceride levels [52-54], while blood glucose levels are consistently lowered [52,53] and plasma ketone body levels are increased [55,56]. In mice, calorie restriction leads to either a decrease or no change in plasma total cholesterol and triglyceride levels [35,57]. Calorie restriction in mice generally leads to a decrease in plasma glucose levels and increase in plasma ketone body levels [13,18,57,58], although there are reports showing glucose levels can remain unchanged with calorie restriction [35], and ketone body levels remaining unchanged or even decreasing with calorie restriction [35,59].

To assess the effects of diet on plasma glucose and ketone levels, along with their effect on hormones and lipids, we evaluated the influence of a standard mouse chow diet, a ketogenic diet, and a fish-oil supplemented diet under *ad libitum* feeding conditions in the inbred C57BL/6J (B6) mouse strain. These diets can be used to treat diseases and disorders, and it remains unclear if the therapeutic efficacy of the diets is related to their composition or to a modest calorie reduction [60,61]. The three diets were also evaluated under calorie-restricted conditions. These diet types have been used extensively in the literature, with the ketogenic diet being used to treat epilepsy and other neurological disorders and the fish-oil supplemented diet being used to treat cardiovascular dysfunction and inflammatory disease [1,62-66]. We found differences in plasma metabolite profiles between the standard diet, ketogenic diet, and fish-oil supplemented diet, when fed *ad libitum*. However, these differences were mostly minimized when administered under calorie restriction, which suggests that biomarkers of health are improved in calorie-restricted diets regardless of macronutrient composition.

## Methods

### Mice

The C57BL/6J (B6) mice were obtained originally from Jackson Laboratory (Bar Harbor, ME). The mice were maintained in the Boston College Animal Care Facility. Adult male mice (120 days of age) were used and housed individually in a temperature-regulated room at 22°C and kept on a 12-hour light–dark cycle. Food was provided either *ad libitum* (AL) or restricted to reduce body weight by 20% (CR). Water was provided *ad libitum* to all mice. All animal procedures were in strict accordance with the NIH Guide for the Care and Use of Laboratory Animals and were approved by the Boston College Institutional Animal Care and Use Committee (IACUC).

### Diets

All mice were fed *ad libitum* with standard rodent chow (Prolab RMH 3000; PMI LabDiet, Richmond, IN, USA) during the first seven days of the study (pre-trial period). This is the standard mouse pellet diet, which contains a balance of vitamins and minerals. The standard diet (SD) was prepared by mixing 1 g of powdered standard mouse diet with 1 mL of water to form a paste. The lard-based ketogenic diet (KD) was prepared by the manufacturer (Zeigler Bros. Inc., Gardners, PA, USA), and has a full complement of vitamins and minerals prepared specifically for rodents. The fish-oil supplemented diet (FO) was prepared by mixing 3 g of powdered standard mouse diet with 1 g of CVS Fish Oil to form a crude paste. The composition of the diets is given in Table 1. All diets were placed into 25 mL beakers, which were previously filled halfway with solid baseplate wax, to allow mice free access to the food.

**Table 1:** Composition (%) of standard diet, ketogenic diet, and fish-oil supplemented diet

**Components**	**Standard diet (SD)**	**Ketogenic diet (KD)**	**Fish-oil diet (FO)**
Carbohydrate	52	1	39
Fat	12	70	34
Protein	23	13	17
Fiber	4	11	3
Ash	6	3	4
Moisture	3	2	3
Metabolizable Energy (kcal/g)	3.20	6.35	4.65

### Dietary treatment

After the seven-day pre-trial period, mice were assigned to one of six groups (n = 4 mice/group): 1) standard mouse chow diet fed AL (SD-UR), 2) standard mouse chow diet fed CR to achieve a 20% body weight reduction (SD-R), 3) the lard-based ketogenic diet fed AL (KD-UR), 4) the lard-based ketogenic diet fed CR to achieve a 20% body weight reduction (KD-R), 5) the fish-oil supplemented diet fed AL (FO-UR), and 6) the fish-oil supplemented diet fed CR to achieve a 20% body weight reduction (FO-R).

Body weights and food intake measurements were taken daily three hours after lights-on. Based on food intake and body weight during the pre-trial period, food in the CR groups was restricted to achieve a 20% reduction in body weight. We used body weight as the endpoint for CR, as we showed previously that body weights are a more stable and consistent variable than food intake, which changes significantly on a daily basis in *ad libitum* fed mice [18,57,58].

The study period lasted a total of 32 days. After the 7-day pre-trial period, mice were fasted for 16 hrs on day 0, before initiating their respective diets. By day 7 of the dietary treatment, body weight and food intake stabilized. Therefore, all calculations involving body weight and food intake were taken from days 7–32 of the study. Metabolizable energy intake was calculated according to the manufacturers’ measurements of metabolizable energy content (Table 1).

### Collection of plasma

After a 3-hr fast on day 32 (during the light cycle), mice were anesthetized with isoflurane, and plasma was obtained by collecting blood into heparinized tubes through the retro-orbital sinus. Plasma was collected by centrifuging blood at 6,000 × g for 10 minutes at 4°C and was stored at -80°C until analysis.

### Measurement of glucose, β-hydroxybutyrate, and hormones

Glucose was measured spectrophotometrically using the Trinder Assay (Sigma-Aldrich, St. Louis, MO, USA). β-hydroxybutyrate was measured enzymatically using a modification of the Williamson *et al.* procedure [67]. Plasma insulin was measured by rat/mouse insulin ELISA (Millipore, Billerica, MA, USA), and plasma adiponectin was measured by mouse adiponectin ELISA (Millipore).

### Measurement of lipids

We analyzed the plasma content of the triglycerides, cholesterol, free fatty acids, cholesteryl ester, phosphatidylcholine, lyso-phosphatidylcholine, phosphatidylethanolamine, and sphingomyelin, as previously described [68]. Briefly, lipids were extracted by adding chloroform (C) and methanol (M) to plasma in a ratio of 30:60:8 (C:M:plasma by volume). The lipid extract was added to DEAE-Sephadex (A-25, GE Healthcare, Piscataway, NJ) to separate the neutral and acidic lipid fractions, as previously described [69]. Neutral lipids were eluted from the column with C:M:dH_2_O at 30:60:8 by volume, and dried by rotary evaporation. The acidic lipids, which contain a portion of the free fatty acids, were eluted with C:M:0.8M sodium acetate at 30:60:8 by volume, and dried by rotary evaporation.

To quantify the lipids, we spotted an equivalent of 3 μL of plasma per lane on 10 × 20 cm Silica gel 60 HPTLC plates (Merck, Darmstadt, Germany) using a Camag Linomat II auto-TLC spotter (Camag Scientific Inc., Wilmington, NC, USA). The plates were developed in a solvent system that contained C:M:acetic acid:formic acid:dH_2_O (70:30:12:4:2 by volume) up to 4.5 cm, and then run in a second solvent system containing hexane:isopropyl ether:acetic acid (65:35:2 by volume) up to 10 cm, as previously described [57,69]. Lipid bands were visualized by charring with 3% cupric acetate in 8% phosphoric acid solution and were scanned using the Personal Densitometer SI (Molecular Dynamics, Sunnyvale, CA, USA). The concentration of each individual lipid was calculated from a standard curve. To measure free fatty acids, which separate into both neutral and acidic lipid fractions, an equivalent of 3 μL of plasma from the neutral and acidic lipid portion was combined and spotted, on a separate plate, as described above.

### Statistical analyses

Group size was determined through power analysis using G*Power 3 statistical software [70]. α error probability and β error probability were set to a maximum of 0.01, and effect size was conservatively estimated from previous data [18,35,57]. This yielded an n of 4 mice per group for six groups, with an actual power of 0.9927 and a critical F value of 4.248. All other statistical analyses were performed using SPSS Software (IBM SPSS Statistics, Version 20). All values are presented as mean ± standard error of the mean (SEM). One-way ANOVA was used to evaluate differences between dietary groups. Bivariate linear regression analysis with ANOVA was used to independently assess the predictive strength of select independent variables on final body weight, plasma glucose, β-hydroxybutyrate, and triglycerides, regardless of dietary treatment. Pearson bivariate correlation analysis was used to determine the relationship between body weight, food intake, plasma glucose, β-hydroxybutyrate, insulin, adiponectin, and lipid levels.

## Results

Dietary modification as a treatment modality is subject to variability, as investigators often use different approaches to implement the diets, which hinders study reproducibility. To improve reproducibility and evaluate the merits of each diet, we fasted all of the mice for 16 hrs prior to initiating the diets. This allows for a proper evaluation of the diets, as the mice will have the same metabolic set point. Also, the influence of the previously administered standard rodent chow on the animal’s metabolism will be minimized [71]. We have also observed that fasting mice before initiating diets also serves to limit the amount of self-restriction that mice impose when switched to an unfamiliar diet (personal observations).

### Body weight and metabolizable energy intake

**Figure 1:**
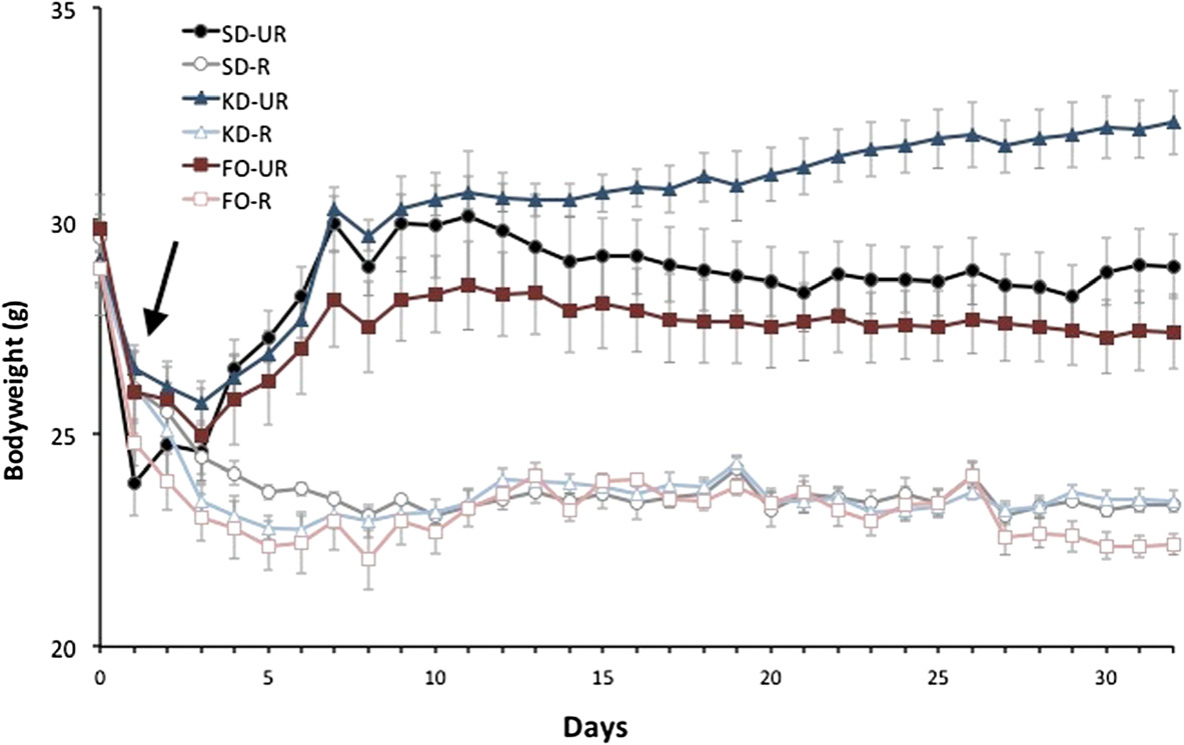
**Influence of dietary regimen on body weight.** Weights are expressed as means ± SEM. Arrow represents initiation of respective diets after a 16 hour fast. *SD-UR*, standard diet unrestricted; *SD-R*, standard diet restricted; *KD-UR*, ketogenic diet unrestricted; *KD-R*, ketogenic diet restricted; *FO-UR*, fish-oil supplemented diet unrestricted; *FO-R*, fish-oil supplemented diet restricted.

All CR mice lost approximately 20% of their initial body weight (Figure 1, Table 2). All mice tolerated the diets well and appeared healthy and vigorous at the end of the study. Activity levels were comparable in all groups, and lethargy was not present in any mouse.

**Table 2:** Influence of dietary intake on bodyweight and calorie consumption in C57BL/6J mice

	**Diet**					
	**Standard diet**		**Ketogenic diet**		**Fish-oil diet**	
	**UR**	**R**	**UR**	**R**	**UR**	**R**
Final Bodyweight (g)^a^	29.0 ± 0.7	23.6 ± 0.1	32.5 ± 0.7	23.3 ± 0.3	27.9 ± 0.9	23.1 ± 0.2
Bodyweight Change (%)	+0.3 ± 1.8	-21.1 ± 1.4	+11.2 ± 2.3	-19.9 ± 0.6	-10.7 ± 2.7	-22.4 ± 1.1
Energy Intake (kcal)^b^	15.0 ± 0.4	11.3 ± 0.6	13.4 ± 0.3	10.1 ± 0.3	13.1 ± 0.6	10.2 ± 0.2
Calorie Difference (%)^c^ from UR Diet	0.0 ± 1.3	-24.4 ± 1.1	0.0 ± 1.2	-24.9 ± 2.2	0.0 ± 4.6	-22.3 ± 1.9
Calorie Difference (%)^d^ from SD-UR	0.0 ± 1.3	-24.4 ± 1.1	-10.5 ± 1.1	-32.8 ± 1.9	-12.2 ± 4.0	-31.8 ± 1.7

^a^Values are expressed as the mean ± SEM (n = 4 mice per group).

^b^Mean metabolizable energy (kcal) consumed per mouse per day from days 7-31.

^c^From UR diet.

^d^From SD-UR.

Body weights stabilized after approximately 7 days following diet implementation (Figure 1), whereas calorie intake stabilized after 8–10 days (data not shown). While the SD-UR group maintained body weight throughout the study, the KD-UR group gained approximately 11% body weight during the 32-day period, despite consuming approximately 10% less in calculated daily calories compared to SD-UR (Table 2). The FO-UR group lost approximately 11% body weight during the 32-day period, and this occurred with a 12% reduction in calorie intake compared to SD-UR. While body weight was reduced by 20% in each CR group relative to the *ad libitum* control group, the amount of CR necessary to achieve this weight loss depended on the macronutrient composition of the diet. Mice fed the higher-fat diets (KD-R and FO-R) required a calorie reduction of approximately 33%, whereas mice fed the normal chow diet (SD-R) required a 24% calorie reduction to achieve the 20% body weight loss. The FO-UR group consumed 700 mg of fish oil per day, whereas the FO-R group consumed 550 mg per day.

### Influence of diet on plasma metabolites and hormones

Glucose levels were reduced in all three CR groups compared to the levels in the SD-UR group and in their respective unrestricted diets (Table 3). Glucose levels were also lower in the FO-UR group than in the SD-UR group, though this might reflect the differences in body weights. The blood glucose levels in the FO-UR group were similar to those in the KD-R group. Blood glucose levels were significantly higher in the KD-UR group than in the SD-UR group.

**Table 3:** Influence of dietary intake on plasma metabolites, hormones, and lipids in C57BL/6J mice

	**Diet**					
	**Standard diet**		**Ketogenic diet**		**Fish-oil diet**	
	**UR**	**R**	**UR**	**R**	**UR**	**R**
**Metabolites**^**a**^						
Glucose (mM)	12.8 ± 0.5	6.1 ± 0.4*	15.1 ± 0.4^§^	8.5 ± 0.5*^§^	8.7 ± 0.6^§^	6.0 ± 0.1*^§^
β-Hydroxybutyrate (mM)	0.4 ± 0.0	1.4 ± 02*	1.1 ± 0.1^§^	2.9 ± 0.2*^§^	0.9 ± 0.1^§^	2.0 ± 0.1*^§^
Glucose/β-Hydroxybutyrate Ratio	35.4 ± 4.7	4.6 ± 1.0*	13.4 ± 2.5^§^	2.9 ± 0.9*^§^	10.4 ± 2.2^§^	3.0 ± 0.3*^§^
**Hormones**^**a**^						
InsuIin (pmoI/L)	182 ± 17	108 ± 3*	160 ± 7	110 ± 4*^§^	112 ± 1^§^	98 ± 2^§^
Adiponectin (μg/mL)	9.2 ± 04	18.9 ± 3.8*	11.1 ± 1.2	15.3 ± 0.8	8.7 ± 0.3	13.8 ± 1.0
**HOMA-IR**^**b**^	15.0 ± 1.6	4.1 ± 0.5*	15.5 ± 0.8	5.7 ± 0.4*^§^	6.2 ± 0.4^§^	3.6 ± 0.1^§^
**Lipids (mg/dL)**^**a**^						
Triglyceride	31.5 ± 3.2	5.0 ± 0.8*	45.5 ± 0.3^§^	7.3 ± 0.2*^§^	8.4 ± 2.3	7.3 ± 1.0^§^
Cholesterol	28.3 ± 2.0	15.9 ± 2.8*	45.9 ± 2.6^§^	30.3 ± 0.3*	22.8 ± 0.5	26.0 ± 2.8
Free fatty acid	42.7 ± 3.4	64.6 ± 4.5*	48.1 ± 3.5	57.1 ± 2.5*^§^	41.2 ± 2.4	44.9 ± 0.9
Cholesteryl Ester	32.1 ± 0.9	26.7 ± 0.5	55.8 ± 0.8^§^	41.0 ± 1.4^§^	31.9 ± 1.3	34.5 ± 3.4
Phosphatidylcholine	64.2 ± 3.8	42.2 ± 2.1*	107.9 ± 4.9^§^	68.8 ± 2.9*	52.7 ± 2.3^§^	59.5 ± 4.4
Lyso-phosphatidylcholine	9.3 ± 0.3	5.5 ± 0.5*	10.6 ± 0.3	6.7 ± 02	6.6 ± 0.6	5.7 ± 0.7
Phosphatidylethanolamine	4.0 ± 0.5	2.6 ± 0.4*	8.2 ± 0.9^§^	5.6 ± 0.1^§^	2.2 ± 0.4^§^	3.0 ± 0.0
Sphingomyelin	6.8 ± 0.3	3.9 ± 2.0*	10.3 ± 0.9^§^	10.2 ± 0.4^§^	6.9 ± 0.1	8.2 ± 0.9

^a^Values are expressed as the mean ± SEM (n = 4 mice per group).

^b^Homeostatic Model Assessment of Insulin Resistance, calculated as (fasted glucose (mM) × fasted insulin (pU/mL)/22.5.

*Indicates significance from unrestricted diet at *p* <0.01 as determined by one-way ANOVA.

^§^Indicates significance from unrestricted standard diet at *p* <0.01 as determined by one-way ANOVA.

β-hydroxybutyrate (major circulating ketone) levels were increased in all the CR groups compared to their respective *ad libitum* diet groups, with the KD-R group exhibiting the highest levels of ketones (Table 3). KD-UR and FO-UR groups had significantly elevated ketone levels compared to the SD-UR group.

Plasma insulin levels were assessed from the mice after a 3-hr fast, which along with fasting blood glucose levels comprise a homeostatic model assessment of insulin resistance (HOMA-IR) value and is indicative of insulin sensitivity [72-74]. Low HOMA-IR values indicate increased insulin sensitivity. Insulin levels were lower in all CR groups than in their comparable *ad libitum* groups, which indicates high insulin sensitivity (Table 3). While insulin levels were similar in the SD-UR and KD-UR groups, insulin levels were lower in FO-UR group and comparable to those in the CR groups. The high insulin sensitivity mirrored the lower plasma glucose levels in FO-UR mice. We found that plasma glucose and insulin levels were highly correlated across all groups (Table 4). Adiponectin levels were highest in the CR groups, with SD-R and KD-R having significantly elevated levels, and FO-R trending toward increased levels (Table 3). The levels of adiponectin were similar across all unrestricted diets.

**Table 4:** Pearsons Bivariate Correlations of bodyweight, macronutrient intake and plasma metabolites

	**Bodyweight**	**Calorie Intake**	**Fat Intake**	**Carbo-hydrate Intake**	**Glucose**	**Ketone**	**Insulin**	**Adipo-nectin**	**Tri-glyceride**	**Cholesterol**	**Free fatty acids**	**Cholesteryl Ester**	**Phos-phatidyl-choline**	**Lyso-phos-phatidyl-choline**	**Phosphatidyl-ethanolamine**
Calorie Intake	.751**														
Fat Intake	.494*	.024													
Carbohydrate Intake	.004	.496*	-.725**												
Glucose	.922**	.707**	.601**	-.019											
Ketone	-.610**	-.860**	.266	-.619**	.478*										
Insulin	.677**	.687**	.319	.202	.745**	-.495*									
Adiponectin	-.640**	-.523**	-.318	.107	-.611**	.414*	-.282								
Triglyceride	.863**	.653**	.568**	-.006	.940**	-.477*	.684**	-.597**							
Cholesterol	.654**	.262	.837**	-.522**	.772**	-.005	.536**	-.469*	.811**						
Free Fatty Acids	-.194	-.265	.099	-.144	-.168	.175	-.077	.123	-.200	-.142					
Cholesteryl Ester	.560**	.079	.922**	-.673**	.667**	.133	.372	-.396	.704**	.944**	.009				
Phosphatidylcholine	.358	.117	.437*	-.317	.401	.004	.306	.400	.480*	.674**	-.164	.550**			
Lyso-phosphatidylcholine	.843**	.661*	.588**	-.020	.939**	-.437*	.772**	-.537**	.957**	.834**	-.221	706**	.536**		
Phosphatidyl-ethanolamine	.555**	.122	.880**	-.500*	.690**	.150	.481*	-.303	.734**	.916**	-.083	.903**	.666**	.755**	
Sphingomyelin	.250	-.087	.722**	-.691**	.405	.377	.324	-.209	.370	.814**	-.160	.760**	.621**	.492*	.734**

*p < 0.05, two tailed t test.

**p < 0.01, two tailed t test.

### Influence of diet on plasma lipids

**Figure 2:**
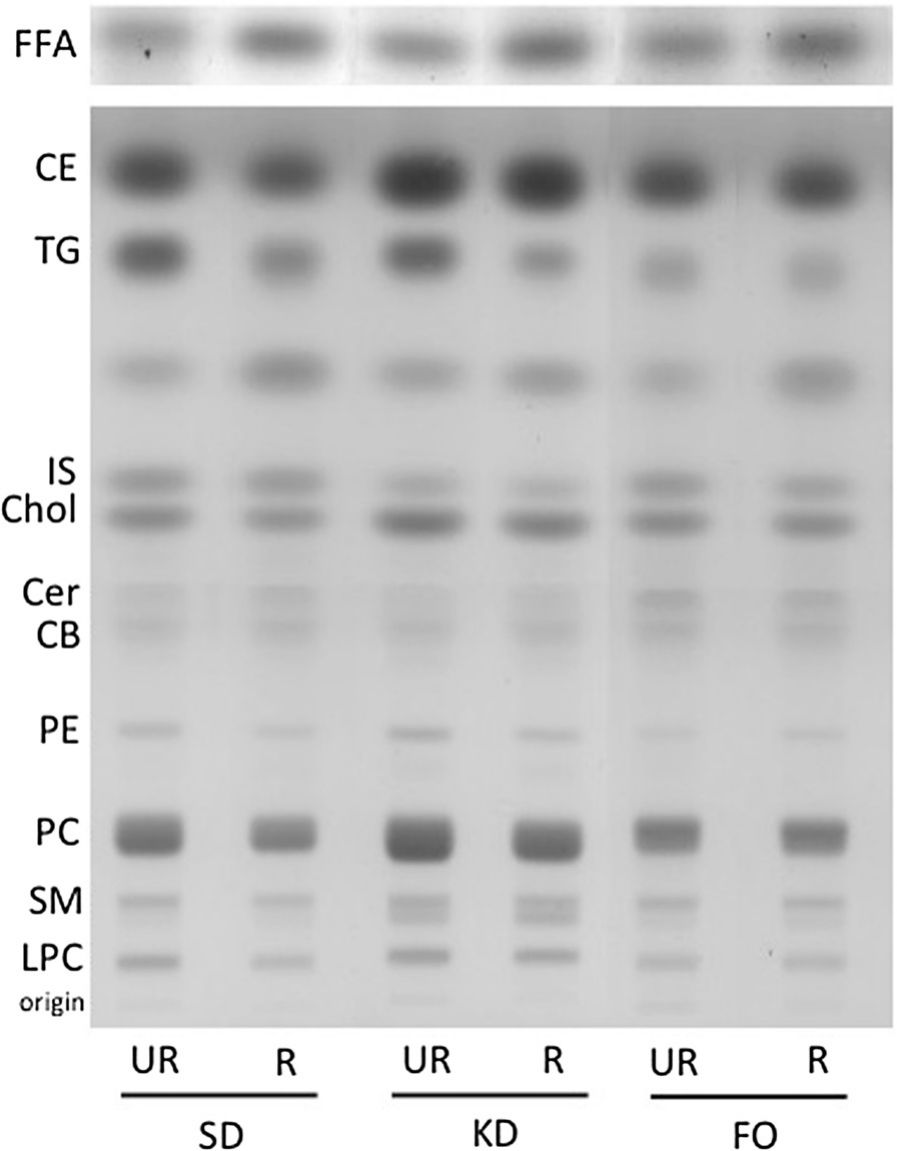
**HPTLC plate of plasma lipids in mice under Standard Diet (SD), Ketogenic Diet, and Fish-Oil Diet (FO) under both unrestricted (UR) and restricted (R) feeding conditions.***FFA*, free fatty acids; *CE*, cholesteryl ester; *TG*, triglycerides; *IS*, internal standard (oleyl alcohol); *Chol*, cholesterol; *Cer*, ceramide; *CB*, cerebrosides; *PE*, phosphatidylethanolamine; *PC*, phosphatidylcholine; *SM*, sphingomyelin; *LPC*, lyso-phosphatidylcholine. Lipids were run on two separate plates (neutral lipids and free fatty acids), as described in the methods. All lipid values are quantified in Table 3, except for ceramides and cerebrosides, since there are only trace amounts present.

We examined plasma lipid distribution using HPTLC (Figure 2). The changes in plasma triglyceride levels were generally correlated with the changes in plasma glucose levels, with the CR groups exhibiting the lowest levels of circulating triglycerides and glucose (Table 3). The KD-UR group had the highest levels of triglycerides, whereas the FO-UR group exhibited triglyceride levels that were similar to the levels measured in the CR groups. Total free cholesterol levels were reduced in the SD-R and KD-R groups compared to their unrestricted controls, although cholesterol levels in the KD-R group were similar to those in the SD-UR group. The cholesterol levels in both fish-oil groups were similar to those seen in the SD-UR group. Cholesteryl ester levels follow a similar pattern to free cholesterol. While cholesteryl ester levels were not significantly decreased in the SD-R compared to the SD-UR, the KD groups had the highest levels of cholesteryl esters, with the KD-UR having significantly higher levels compared to the KD-R. Cholesteryl ester levels were unchanged in the fish-oil groups, and were similar to those in the SD-UR group. Free fatty acid levels (FFA) were higher in the SD-R and KD-R groups than in their respective UR groups.

Phosphatidylcholine (PC) levels were reduced in the SD-R and KD-R groups compared to their respective unrestricted diets, but were higher in the KD group. The fish-oil groups had similar PC levels. Lyso-phosphatidylcholine (LPC) levels were reduced slightly in the SD-R group, and were similar across the rest of the groups (Table 3). Plasma phosphatidylethanolamine (PE) levels were lowest in the fish-oil groups, and were significantly reduced in the SD-R and KD-R groups compared to the unrestricted diets, although the KD groups had the highest overall levels of PE. Sphingomyelin (SM) levels were highest in the KD groups, and were significantly reduced in the SD-R compared to the SD-UR. There was no difference between the fish-oil groups, and they were comparable to the levels seen in the SD-UR. Plasma ceramide and cerebroside levels were also evaluated, however they were present only in trace amounts and were not quantified (Figure 2).

### Major Predictors of body weight, glucose, ketone, and triglyceride levels

**Figure 3:**
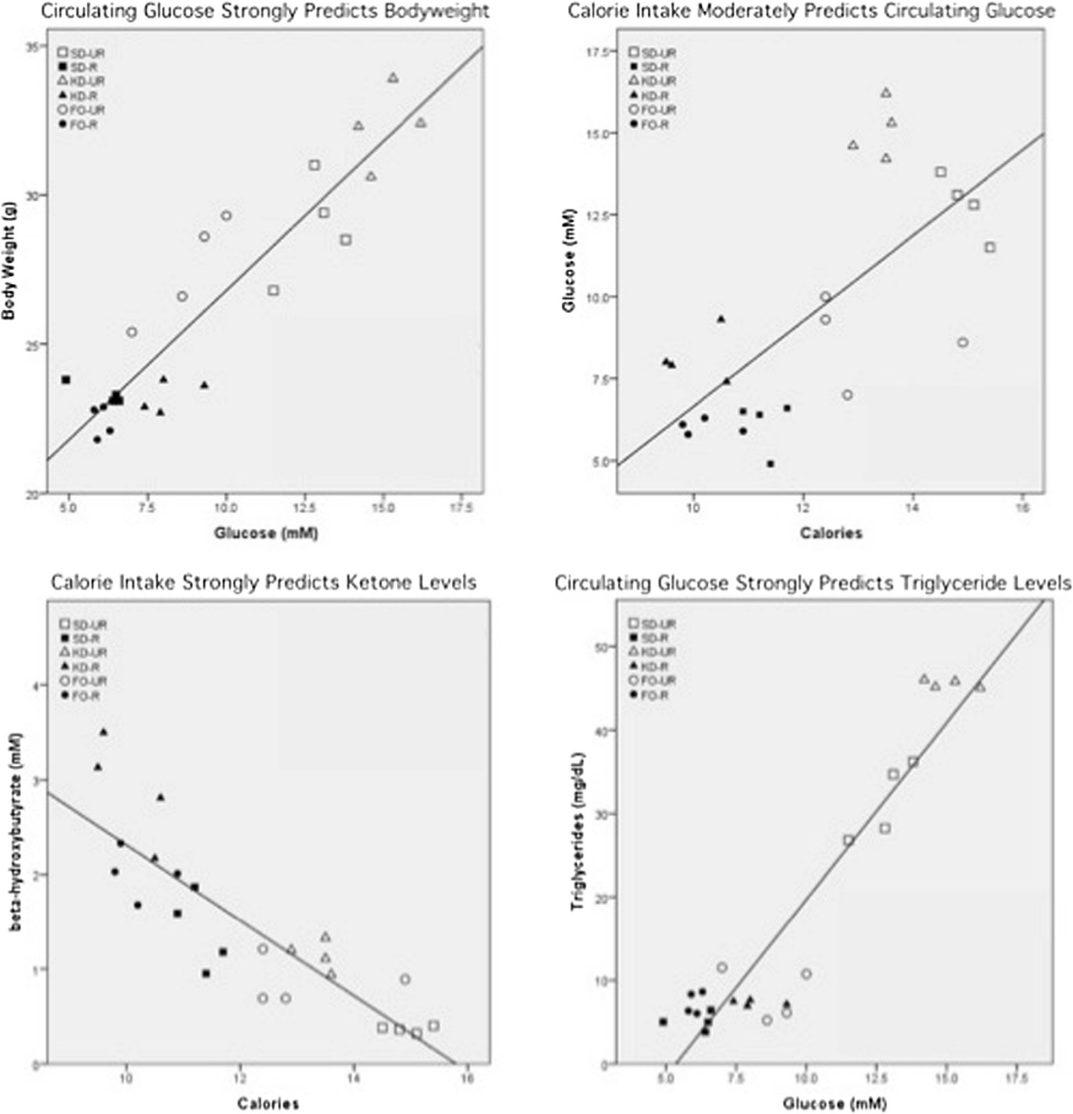
**Linear regression analyses of body weight, glucose, β-hydroxybutyrate, and triglyceride levels in mice.** The strongest predictor (highest coefficient of determination) for each dependent variable is plotted. Irrespective of diet, glucose levels most strongly predict body weight and triglyceride levels, calories most strongly predict glucose and ketone levels, and dietary fat intake most strongly predicts cholesterol levels. *SD-UR*, standard diet unrestricted; *SD-R*, standard diet restricted; *KD-UR*, ketogenic diet unrestricted; *KD-R*, ketogenic diet restricted; *FO-UR*, fish-oil supplemented diet unrestricted; *FO-R*, fish-oil supplemented diet restricted.

We pooled the dietary groups to independently assess the predictive strength of independent variables (Figure 3). The assumptions of bivariate linear regression with ANOVA were met, according to established criteria [75]. The strongest independent predictor (highest coefficient of determination) for final body weight, plasma glucose, ketone, and triglyceride levels is displayed in Figure 3 for each of the variables assessed. We found that while calories and ketones moderately predict body weight, blood glucose level (R^2^ = 0.850; 95% CI = 1.000 ± 0.186; *t*_22_ = 11.157; *y* = 16.798 + 1.000*x*) was the strongest predictor of final body weight during the study. The amount of dietary fat and dietary carbohydrates consumed were not significant indicators of final body weight.

While we found that circulating glucose levels were the strongest predictor of final body weight, calorie intake was the most significant predictor of blood glucose level (R^2^ = 0.500; 95% CI = 1.304 ± 0.5777; *t*_22_ = 4.687; *y* = -6.390 + 1.304*x*). Dietary fat intake also moderately predicted plasma glucose levels, whereas dietary carbohydrate intake was not a significant predictor of glucose levels.

The most important predictor of circulating ketones was reduced calorie intake (R^2^ = -0.740; 95% CI = -0.399 ± 0.105; *t*_22_ = -7.904; *y* = 6.301-0.399*x*). While lowering calories had the greatest effect on increasing ketone levels, lowering dietary carbohydrate intake also had a significant, but moderate influence (R^2^ = 0.384; 95% CI = -0.591 ± 0.332; *t*_22_ = -3.700; *y* = 2.018-0.591*x*) on ketone levels. Glucose level was a weak predictor of ketone levels, and surprisingly, dietary fat intake was not a significant predictor of circulating ketone levels.

Circulating glucose levels strongly predicted circulating triglyceride levels (R^2^ = 0.883; 95% CI = 4.223 ± 0.672; *t*_22_ = 12.870; *y* = -22.631 + 4.233*x*). Calorie and dietary fat intake moderately predicted triglyceride levels, whereas ketone levels were a weak predictor of triglyceride levels. Dietary carbohydrate intake was not a predictor of circulating triglyceride levels.

## Discussion

We found that the macronutrient composition of a diet greatly affected the plasma metabolite profile of C57BL/6J mice when administered *ad libitum*. A ketogenic diet administered *ad libitum* promoted high glucose levels, weight gain, and a hyperlipidemic profile in mice, whereas fish-oil supplementation decreased bodyweight, glucose levels, and yielded a normolipidemic profile. Administration of these diets under CR led to weight loss, increased insulin sensitivity, decreased glucose levels, increased ketone levels, and promoted a normolipidemic lipid profile. When administered under CR, the influence of the composition of the diet on the metabolic profiles of the mice was minimized, yielding similar metabolic profiles across all groups. Thus, a restriction of total energy intake can correct weight gain or a hyperlipidemic profile from these dietary therapies in B6 mice.

We confirmed previous studies that the KD-UR does not lead to long-term weight loss in mice when they are fasted prior to diet initiation [13,18,76]. Our work supports the notion that “a calorie is not a calorie”, as the KD-UR group gained approximately 10% body weight despite consuming 10% less in metabolizable calorie intake, compared to the SD-UR. However, our results challenge the metabolic advantage theory of the KD-UR, which states that isocaloric diets low in carbohydrates lead to greater weight loss compared to isocaloric diets of different composition [77]. This was unsurprising, given that dietary fat is metabolized more efficiently than are dietary carbohydrates and proteins [78]. The seeming discrepancy between metabolizable calorie intake and body weight is most likely due to differences in energy utilization between the different dietary groups, including factors such as obligatory thermogenesis, microbial fermentation, diet induced thermogenesis, and basal metabolism (including energy expenditure) [79].

In addition to the increased body weight under the KD-UR, the plasma profile of the KD-UR group suggests that the diet is unhealthy [80-83]. The plasma profile of the KD-UR mice is similar to a previously published report utilizing a high-fat, high-sucrose diet in the same strain of mice [35]. The atherogenic lipids triglycerides, cholesterol, and cholesteryl esters were highest in KD-UR group. A KD-UR diet also elevated these lipids in children with epilepsy [28]. The presence of high circulating glucose levels in the KD-UR group, which has also been reported in other studies of rodents on high-fat, low-carbohydrate diets [84,85], could further increase the risk of atherogenesis, as glucose can contribute to endothelial dysfunction [86,87]. Glucose can be raised in high-fat diets devoid of carbohydrates with excess calorie intake through gluconeogenesis, which occurs indirectly through oxidation of fatty acids to acetyl-CoA [88]. The KD-UR group also had the highest levels of PC, LPC, PE, and SM. It has been suggested that a PC to free cholesterol ratio (mol/mol) of less than 1 is a risk factor for atherosclerosis [89]. The calculated PC/cholesterol ratios in all of the groups (both restricted and unrestricted) were similar, and ranged from 1.5-1.75 (data not shown). LPC levels have been shown to be positively associated with obesity [90]. PE levels are suggested to be positively associated with atherosclerotic complications [91]. SM in plasma is associated with an increased risk of coronary artery disease [92,93]. Our data are consistent with the observations for LPC, PE, and SM. These findings, together with our observations in B6 mice, indicate that consumption of the KD in unrestricted amounts can have adverse effects on health-related biomarkers.

Administration of the KD in restricted amounts mitigated the hyperlipidemic plasma profile of the B6 mice. In addition to lowering triglycerides, cholesterol, phospholipids (PC, LPC, and PE), and glucose, the KD-R also lowered fasting insulin levels. Reduced levels of circulating insulin is antiatherogenic [94]. Plasma ketone levels were also highest in the KD-R group. Ketones are therapeutic against a variety of neurological diseases and cancer [1,48,95-97]. It is well known that ketones can replace glucose as an energy metabolite and can protect the brain from hypoglycemia [16,98,99]. Our previous findings, in conjunction with the results in the present study, suggest that the therapeutic efficacy of the KD for epilepsy and brain cancer will likely be best when the diet is administered in restricted than unrestricted amounts [13,18,20].

While the KD-UR increased body weight and was associated with a hyperlipidemic plasma profile, we were surprised to find that the FO-UR diet caused changes in body weight and plasma metabolites that were similar to those seen in the CR groups. We cannot completely rule out that these results are a consequence of decreased food palatability in the FO-UR group, as the FO-UR group had reduced calorie intake compared to the SD-UR. The FO-UR group, however, consumed a similar number of calories as the KD-UR group after the fast (16.9 ± 2.3 (FO-UR) calories versus 17.0 ± 0.3 (KD-UR) calories) and throughout the study, and did not have observable aversion to the food. We hypothesized that increased levels of the adipokine adiponectin might be responsible for the increased insulin sensitivity and decreased body weight in the FO-UR group despite feeding *ad libitum*, since supplementation of fish-oil is associated with increased levels of adiponectin [100,101]. Interestingly, we found that adiponectin was not elevated in the FO-UR group, suggesting that adiponectin by itself was not responsible for the increase in insulin sensitivity or the CR-like profile of the FO-UR mice. Omega-3 fatty acids and their metabolites can produce changes in the activity of peroxisome proliferator-activated receptor (PPAR) α and γ, along with directly binding to and modulating the activity of sterol receptor element binding proteins (SREBP), which contribute to modulating lipid and glucose metabolism [102,103]. Omega-3 fatty acids and their metabolites (epoxide derivatives, docosanoids, resolvins, and neuroprotectins) reduce triglyceride levels, adiposity, and inflammation while improving insulin sensitivity in rodents and humans [104,105]. Thus, the observed plasma profile of the FO-UR mice that mimicked the CR groups were likely due to a multitude of mechanisms regulated by omega-3 fatty acids that are still not fully elucidated.

All of the dietary groups were combined in a post-hoc analysis to examine the relationship among body weight, dietary intake, and plasma profile. Our findings indicated that blood glucose was the strongest predictor of body weight in mice and was highly correlated with body weight. This observation is consistent with previous studies in humans, though the human studies did not review dietary intake [106,107]. We found that intake of specific macronutrients was not predictive of final bodyweight. This observation questions the utility of advocating for diets devoid in specific macronutrients to control body weight [7,108]. In terms of predicting glucose levels, total calorie intake was only moderately predictive suggesting that multiple factors modulate blood glucose levels in mice. Our findings suggest that restriction of total calorie intake will be more effective for reducing glucose levels and body weight in mice than will be the restriction of any particular macronutrient under *ad libitum* feeding. The reduction of total calories was strongly predictive of elevated ketone levels. Reduced carbohydrate intake contributed moderately to increased ketone levels. From a physiological perspective, given that the body has a large reservoir of lipid stores and a small reservoir of glycogen stores, it should not be surprising that restriction of energy intake and carbohydrates will quickly diminish glycogen stores, leading to beta-oxidation of lipids for energy [109]. Dietary strategies that promote beta-oxidation of lipids, through either restriction of overall energy intake or severe carbohydrate restriction, would promote ketosis. Since circulating glucose strongly predicted plasma triglyceride levels, dietary strategies that lower blood glucose levels should have significant effects on promoting cardiovascular and overall health [110,111].

## Conclusions

The macronutrient composition of various diets plays an important role when the diet is fed *ad libitum*, as our data showed for body weight, hormones, and plasma metabolites. CR, however, has a dominant and independent effect on body weight, hormones, and plasma metabolites, compared to macronutrient composition. Our data suggest that CR may be more appropriate for improving health outcomes than shifting macronutrient ratios, especially when using dietary therapy to treat metabolic diseases [13,18].

## Abbreviations

SD: Standard diet; KD: Ketogenic diet; FO: Fish-oil supplemented diet; UR: Unrestricted; R: Restricted; AL: *Ad libitum*; CR: Calorie restriction; FFA: Free fatty acids; PC: Phosphatidylcholine; LPC: Lyso-phosphatidylcholine; PE: Phosphatidylethanolamine; SM: Sphingomyelin.

## Competing interests

The authors declare they have no competing interests.

## Authors’ contributions

JJM carried out the diet implementation, hormone analysis, assisted with lipid analysis, performed the statistical analysis, and drafted the manuscript. NT carried out the diet implementation, lipid and metabolite analysis, and helped to draft the manuscript. TNS conceived the study, and participated in its design and coordination and helped to draft the manuscript. All authors read and approved the final manuscript.
